# *RYR2* Sequencing Reveals Novel Missense Mutations in a Kazakh Idiopathic Ventricular Tachycardia Study Cohort

**DOI:** 10.1371/journal.pone.0101059

**Published:** 2014-06-30

**Authors:** Ainur Akilzhanova, Christian Guelly, Omirbek Nuralinov, Zhannur Nurkina, Dinara Nazhat, Shalkhar Smagulov, Azat Tursunbekov, Anar Alzhanova, Gulzhaina Rashbayeva, Ayan Abdrakhmanov, Sholpan Dosmagambet, Slave Trajanoski, Zhaxybay Zhumadilov, Almaz Sharman, Mahabbat Bekbosynova

**Affiliations:** 1 Department of Genomic and Personalized Medicine, Center for Life Sciences, Nazarbayev University, Astana, Republic of Kazakhstan; 2 Center of Medical Research, Medical University of Graz, Graz, Austria, Graz, Austria; 3 National Scientific Cardiac Surgery Center, Astana, Republic of Kazakhstan; Indiana University, United States of America

## Abstract

Channelopathies, caused by disturbed potassium or calcium ion management in cardiac myocytes are a major cause of heart failure and sudden cardiac death worldwide. The human ryanodine receptor 2 (RYR2) is one of the key players tightly regulating calcium efflux from the sarcoplasmic reticulum to the cytosol and found frequently mutated (<60%) in context of catecholaminergic polymorphic ventricular tachycardia (CPVT1). We tested 35 Kazakhstani patients with episodes of ventricular arrhythmia, two of those with classical CPVT characteristics and 33 patients with monomorphic idiopathic ventricular arrhythmia, for variants in the hot-spot regions of the *RYR2* gene. This approach revealed two novel variants; one de-novo *RYR2* mutation (c13892A>T; p.D4631V) in a CPVT patient and a novel rare variant (c5428G>C; p.V1810L) of uncertain significance in a patient with VT of idiopathic origin which we suggest represents a low-penetrance or susceptibility variant. In addition we identified a known variant previously associated with arrhythmogenic right ventricular dysplasia type2 (ARVD2). Combining sets of prediction scores and reference databases appeared fundamental to predict the pathogenic potential of novel and rare missense variants in populations where genotype data are rare.

## Introduction

Ventricular arrhythmias are the leading cause of morbidity and mortality worldwide, causing more than 300,000 sudden cardiac deaths (SCD) each year in the United States and making this a major public health concern [1]. Approximately 5 to 12% of these deaths occur in previously asymptomatic seemingly healthy subjects. In the past few years, the genetic basis of several monogenetically inherited arrhythmogenic syndromes has been discovered, providing novel insights to the molecular understanding of diseases predisposing to SCD [2], [3]. On the basis of this knowledge, it has become apparent that “idiopathic cardiac arrest” may be caused by subclinical or misdiagnosed forms of arrhythmogenic diseases that eluded clinical diagnosis until they unexpectedly manifest with SCD [4]. At least some arrhythmias are caused by one or more mutations in genes that control electrical conduction through the heart by altering calcium homeostasis or depolarization or repolarization gradients in the ventricle. Mutations in these genes can lead to defective cardiac ion channels, causing ventricular arrhythmias [1]–[5].

Since its identification as the major candidate gene in autosomal-dominantly inherited forms of CPVT, more than 180 genetic variants, mostly missense mutations of the *RyR2 gene* have been attributed to arrhythmogenic disorders including CPVT1 (OMIM 604772) and ARVD2 (OMIM 600996) [6], [7]. CPVT is characterized by exercise- or stress-induced ventricular arrhythmia, syncope, or early sudden death, but not at rest. Patients with CPVT have morphologically normal hearts [7]–[9].

Recent work has elucidated that the vast majority of mutations mainly cluster to four regions of the RYR-2 channel encoded by 45 exons [10]. While clusters I-III (I: aa44-466, II: 2246-2534, III: 3778-4201) locate to the large N-terminal cytoplasmic region (aa1-4500) cluster IV (aa4497-4959) locates or to the SR transmembrane region [10], [11]. The localization of the mutations at the various N-terminal, central domain or C-terminal of the protein consistently reported an increased activation of the mutant RYR2 channels to luminal Ca2+ activation [12] with few exceptions. ARVD2 associated RYR2 mutations (p.R176Q, p.L433P, p.N2386I and p.T2504M) locate to two distinct clusters in the cytosolic part of the molecule that correspond to the mutational domains of the skeletal muscle RYR1 channel causing malignant hyperthermia (MH) or central core disease (CCD).

A rarer autosomal-recessive form of inherited CPVT (CPVT2; OMIM 611938) has been associated with homozygous or compound heterozygous mutations in the gene encoding calsequestrin-2 CASQ-2. Mutations in the triadin (TRDN; CPVT5), calmodulin-1 (CALM1; CPVT4) and the inward rectifying potassium channel gene KCNJ2 have also been associated with autosomal dominant forms of CPVT [13], [14]. Another chromosomal locus associated with a severe form of CPVT in a consanguineous family has been mapped to 7p14-p22 (CPVT3) [15].

CPVT has a high mortality rate of up to 50% by the age of 30 years, but since β-blockers and/or implantable cardioverter-defibrillator (ICD) can at least partially prevent arrhythmias and sudden death, early diagnosis through clinical and genetic screening is essential for effective clinical management of patient's suffering from RYR-2 mediated arrhythmia [16]. The introduction of “high-speed” sequencing technologies in modern pathology has highlighted the unprecedented potential to rapidly detect novel genetic variants but at the same time unrevealed the high risk for misclassification in case of rare genetic variants in the past [17]. Despite of the easily accessible and continuously growing number of high-quality exome and genome sequencing databases and improved user-friendly in-silico prediction tools, considerable caution has to be taken to separate genetic noise from truly disease causing rare variants especially in populations where genetic data are rare. In a first pilot study we screened 35 unrelated patients from our Kazakhstani ventricular tachycardia study cohort suffering from ventricular tachycardia (most of them idiopathic) for genetic variants in the mutational hot-spot regions of the RYR2 gene and classified the observed variants. The demographic background of our patient study group is 54.3% (19/35) Kazakh, 34.3% (12/35) Russian, and 11.4% others (Table S1 in File S1).

## Material and Methods

### Study participants

The study was performed under the appropriate institutional ethics approvals and in accordance with the principles embodied in the Declaration of Helsinki. A study protocol was approved by the Ethics Committee of the Nazarbayev University. Informed written consent was obtained from all participants. Participants provided written informed consent to include case details in the paper. A clinical diagnosis of ventricular tachycardia was verified in all patients by the authors (M.B., O.N.) and other physicians of the National Scientific Cardiac Surgery Center (NSCC). Of these patients, 2 were classified as “CPVT phenotype” because of exertional syncope plus documentation of bidirectional or polymorphic ventricular tachycardia (Case #271, Case #239), and 33 were classified as idiopathic ventricular tachycardia (Case #444 and others). The subjects and their relatives were investigated at the NSCC. They underwent basic clinical investigation, and history was focused on possible CPVT symptoms, that is, syncope during exercise, history of sudden cardiac death in the family (Overview patient information is given in Table S1 in File S1.

### DNA sequencing of RYR2 mutational hot-spot exons

Genomic DNA was isolated from EDTA blood samples using QIAGEN blood DNA Mini Kit (QIAGEN, Hilden, Germany). Since previous studies consistently detected disease-associated variants within three distinct hot-spot regions of the *RYR2* gene, which encode critical functional and regulatory domains of the channel, we focused sequence analysis to these 45 exons (3, 8, 10, 12–15, 17, 19, 21, 26–28, 37, 40–50, 75, 83, 86–93, 95–97, 99–105) [10], [18].

We used 20 ng of DNA per PCR in a total volume of 20 µl using Hot Start Plus Polymerase (QIAGEN, Hilden, Germany) and a four primer reaction set-up. The primer-set included 1 µl of 10 pmol M13 forward tagged gene-specific forward, and M13 reverse tagged gene-specific reverse primers each and 0.2 µl of 10 pmol gene-specific forward and reverse primers each (both overlapping to 100% with their M13-tagged counterparts regarding the gene-specific sequence). Primers were re-designed based on published primer sequences [18] and updated according to the latest db entry for the hRYR2 gene (ENSG00000198626; from ENSEMBL data base release GRCh37; date of primer design: Oct. 23^rd^, 2012). Primer sequences are listed in Table S2 in File S1. For all amplicons the same standard PCR cycling conditions were used: 96°C for 5 min followed by 8 cycles of 95°C for 45 sec, 57°C for 30 sec and 72°C for 45 sec, 27 cycles of 95°C for 30 sec, 68°C for 30 sec and 72°C for 45 sec and a final extension step of 72°C for 10 min. PCR products were purified using the Marchery&Nagel PCR clean-up system in an automated setting with a Hamilton Starlet liquid handling robot. Sequencing reactions were run using 3.1. BigDye Terminator chemistry and M13 forward or M13 reverse sequencing primers according to standard cycling conditions, purified and sequenced on an ABI 3730 DNA Sequencer (Life Technologies). Sequence analysis was done both, electronically (DNAStar, Lasergene, SeqMan Tool) with the SNP discovery threshold set to 35% and manually. As reference sequence of the human *RYR2* gene, GenBank entry NM_001035 was used.

### Classification of genetic variants

To predict the possible impact of amino acid substitutions, the following prediction tools were used: 1) SIFT (Sorting Intolerant From Tolerant, v.4.05) [19], 2) PolyPhen-2 (Polymorphism Phenotyping v2) [20], 3) Grantham Score [21], 4) MutationTaster2 [22], and 5) Conservation. In addition, the databases of the 1000Genomes and the Exome Sequencing Project (ESP; n = 6,503) and the Human Genome Mutation Database were queried to further evaluate and/or validate the variants in a second dimension. Additionally, samples of 192 Kazakh individuals (KazCG) and samples of 96 unrelated breast cancer patients from the Kazakh population were used as a control group (Kazakh Breast Cancer Study Cohort (KazBCSC). We classified our missense variants related to a recently proposed classification system [23]. For conservative classification we defined a missense variant as possibly damaging variant when at least three of the five prediction tools predicted the variant damaging and when ESP6500, the 1000Genomes or the Kazakh Control Group (KazCG; n = 192) databases were negative for the variant. With three or less than three “damaging” predictions but at least one positive entry in one of the three databases a variant was called Variant of Unknown Significance (VUS).

## Results

### Case #271

**Figure 1:**
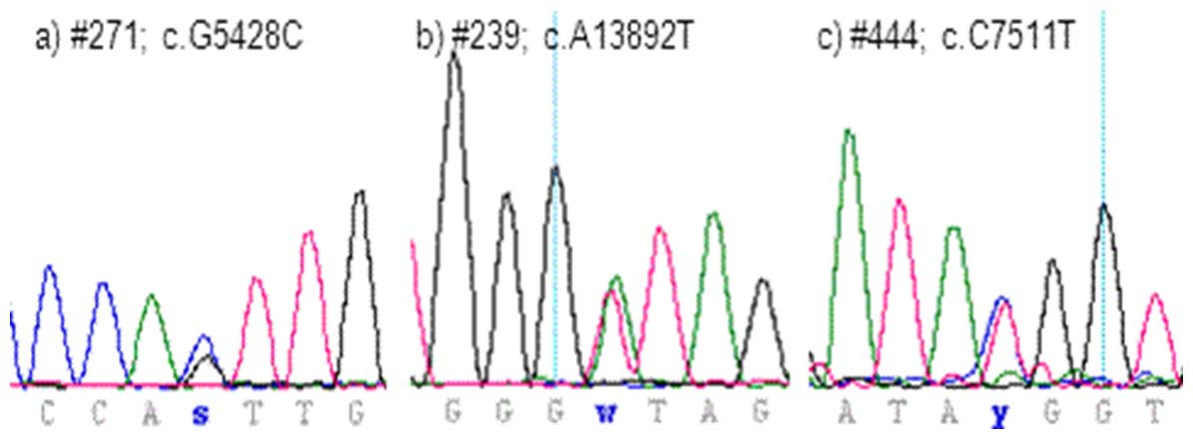
Electropherograms of RYR2 sequences from patients #271 (a), #239 (b), and #444 (c).

We observed missense variant p.V1810L (c5428G>C; Fig.1 a) in a 42 year old male Korean. The initial diagnosis was idiopathic arrhythmia characterized by unstable paroxysms of ventricular tachycardia. The age of VT onset was 41 with negative anamnesis for cases SCD or heart failure in his family. He was admitted to the NSCC with complaints of monotone pains and left hand numbness unrelated physical stress. ECG Holter monitoring fixed episodes of unstable ventricular tachycardia. ECHO-CG did not reveal any structural heart pathologies. Thread mill test was negative and tolerance towards physical stress high. A detailed summary of clinical characteristics and ECG data is shown in Table S1 in File S1 and Figure S1 in File S1.

#### Resting ECG

The ECG heart rhythm at rest was sinusoid with 49 beats/min, a normal QRS axis, a normal PR interval (188 ms), QRS (74) ms and QT (446 ms), QTC (402 ms). There were no ST-T or T wave abnormalities.

#### VT characteristics

At the time of VT onset, the preceding sinus rate at VT onset was 83 beats/min, bigeminy. The VT heart rate was 220 beats/min, CL - 227 ms. VT complexes (n = 14) at onset were non sustained with a duration of paroxysms of 3.2 sec. The VT was monomorphic. Wide QRS-complex tachycardia with elements for diagnosis of VT (AV dissociation), probably originating in the left ventricle were observed. CL is changing depicting warming-up phenomenon (non-reentrant mechanism).

#### RYR2 variant prediction

The genetic variant c5428G>C encoding p.V1810L was identified in patient #271 (Table 1). Three out of five algorithms predicted this variant damaging. In addition, the ESP6500, the 1000Genomes Project and the KazCG were negative for the variant whereas one female patient of the KazBCSC carried the same genetic variant. Unfortunately, the control subject was not available for further clinical examination. The family history of the index patient was negative for cases of sudden cardiac death or cardiac rhythm disturbances. The index patient's asymptomatic mother was tested negative for the variant; the father did not participate in the study.

**Table 1:** List of nonsynonymous RYR2 single nucleotide variants*.

AA Variation	Cod. pos. and nt subs.	SIFT Score	Poly-phen	Mutation Taster	Grantham Score	Conservation	Familial anamnesis, Segregation	Agreement (B<3, 3≤VUS<5, D≥5) Classification
T2504M	c7511C>T	D (0.01)	D (0.99)	D (0.99)	B (82)	Conserved (D)	unknown	D
V1810L	c5428G>C	D (0.03)	B (0.24)	D (0.98)	B (32)	Conserved (D)	inherited; no familial history of SCD or CD	VUS
D4631V	c13892A>T	D (0.0)	D (0.99)	D (0.99)	D (152)	Conserved (D)	De-novo; no familial history of SCD or CD	D

*AA: Amino Acid. D: Damaging. VUS: Variant of Unknown Significance. B: Benign. SCD: Sudden Cardiac Death. CD: Cardiac Death.

### Case #239

Missense variant p.D4631V RYR2 (c13892A>T, Fig. 1 b) was detected in a 23 year old female Kazakh. Age of symptom onset was 13 years with recurrent episodes of syncope and occurrence of characteristic ECG patterns with mono/polymorphic ventricular premature beats followed by bi-directional ventricular tachycardia and salvos of polymorphic ventricular tachycardia.

She was initially admitted to NSCC with complaints about paroxysms of heart palpitation, breathlessness at exercise, weakness and fatigue. She suffered palpitation, dizziness, seizures, episodes of syncope, frequent respiratory infections, chronic pyelonephritis and scoliosis since childhood. At age of 16 CPVT was diagnosed followed by radiofrequency ablation of the right ventricle and the cava tricuspid isthmus due to the high risk of SCD. In the same year a cardioverter defibrillator was implanted and beta blockers administered. During the follow up period and during ICD programming she perceived episodes of presyncope due to ventricular tachycardia of 260 ms cycle length. Ventricular tachycardia was treated by burst stimulation. Family history was negative for episodes of syncope and SCD. A detailed summary of clinical characteristics and ECG data is shown in Table S1in File S1 and Figure S2 in File S1.

#### Resting ECG

The ECG heart rhythm at rest was sinusoid with 60 beats/min, a normal QRS axis, a normal PR interval (166 ms), QRS (96) ms and QT (432 ms), QTC (428 ms). There were no ST-T or T wave abnormalities. The arrhythmias detected at rest were premature ventricular beats and atrial tachycardia.

#### VT characteristics

At the time of VT onset, QRS complex was relatively narrow, at 90 ms. The preceding sinus rate at CPVT onset was 119 beats/min, bigeminy. The CPVT heart rate was 234 beats/min, CL- 260 ms. The recurrent CPVT was polymorphic and bidirectional.

The QRS morphology is characteristic of bidirectional ventricular tachycardia changed every 5–6 beats, typically with inferior and superior axis or right bundle branch block and left bundle branch block patterns. CPVT was induced by exercise. Exercise test was not performed.

Catecholamine infusion test was not performed.

#### Pathogenicity Predication

RYR2 missense variant p.D4631V was predicted pathologic/disease causing by five out of five prediction algorithms (Table 1). The variant was neither reported in the ESP6500, the 1000 Genomes database nor found in the KazCG or the KazBCSC. There was no indication of SCD, heart failure or less severe cardiac disorders in the family history. In the absence of strong co-segregation – both parents were tested negative for the variants - or functional studies the p.D4631V de-novo variant must still be considered as possibly pathogenic.

### Case #444

The variant p.T2504M (c7511C>T) was detected in 20 year old female Kazakh. She was diagnosed with cardiac arrhythmia, unstable ventricular tachycardia. The age of onset of VT was 19 years. She was under outpatient observation of cardiologist for the reason of premature ventricular beats, and was admitted to the NSCC with complaints of shortness of breath at slight physical activity, general weakness, periodic pains and irregular heartbeats. No structural abnormalities were detected by ECHO-CG. The patient was treated with anti-arrhythmic drugs, but they were not effective for premature ventricular contractions and ventricular tachycardia. Thus the radiofrequency ablation of arrhythmogenic focus located in the right ventricular outflow tract was performed twice, the last one was successful and following 24-hour ECG monitoring showed no arrhythmia manifestation. A detailed summary of clinical characteristics and ECG data is shown in Table S1 in File S1 and Figure S3 in File S1.

#### Resting ECG

The ECG heart rhythm at rest was sinusoid with 76 beats/min. The electrical heart axis was at vertical position. PR (140) ms, QRS (86) ms, QT (382 ms), QTC (398 ms). The ST-T waves were normal.

#### VT characteristics

24-hour ECG Holter monitoring revealed 6642 ventricular episodes with morphology of LBBB, where PVBs 600, bigeminy PVBs 332, paired PVBs 1544, paroxysms of VT 192 with maximum of 5 complexes.

The observed missense variant RYR2 p.T2504M (c7511C>T, Fig. 1 c) has been reported in context of ARVD2 (HGMD CM010424) previously [6]. ARVD2 phenotypes mimic and overlap with CPVT phenotypes since structural abnormalities (myocyte loss with fatty or fibro-fatty tissue replacement predominantly of the right ventricle) frequently lack or remain undetected using non-invasive imaging methods. In-silico prediction strongly suggests a disease causing consequence of the mutation (Table 1).

## Discussion

*RYR2* mutations have been attributed to a spectrum of variable and converging clinical phenotypes such as patients with reproducible bidirectional VT and polymorphic VT at exercise stress testing; patients presenting only with polymorphic VT; and patients with idiopathic VT. Even more, the occurrence of CPVT1 and ARVD2 even within the same family raised the suggestion that the two entities might correspond to different degrees of phenotypic expression of the same disease [13]. Considering the various phenotypes and the variable penetrance of the numerous *RYR2* mutations, we thought to investigate 35 patients, 33 of them diagnosed with idiopathic VT for mutations in the *RYR2* gene in a pilot study. We observed a heterozygous missense mutation at c13892A>T (p.D4631V; Case #239) with high pathogenic potential (high in-silico prediction scores, de-novo, reference databases negative) in a patient with classical clinical characteristics of CPVT. The penetrance of this de-novo variant has to be considered high due to the early age of onset and the distinctive and severe clinical course. Due to ongoing symptoms and risk of SCD index patient #239 is considered to undergo the selective sympathectomy. The ARVD2 variant p.T2504M has been observed in a 20-year old patient with idiopathic ventricular tachycardia. Index patient #444 had a successful RFA and anti-arrhythmic drugs need not be prescribed. However, based on the identification of the ARVD2 associated *RYR2* gene mutation, the patient management strategy was modified. MRI of the heart is planned to perform to detect any arrhythmogenic dysplasia.

The third variants (p.V1810L; Case #271) pathogenic impact on RYR2 channel function remains elusive. Late disease onset and lack of other clinical CPVT characteristics argue against a mechanistic association with the phenotype. However, considering the severity of CPVT misdiagnosis, we may have to consider such rare genetic variants as low-penetrance or susceptibility variants predisposing carriers to CPVT-like symptoms under certain conditions unless a neutral effect is proven; if only in functional studies. The variant detected in index patient #271 was found in an asymptomatic offspring of the patient. Clinical evaluation of the offspring did not indicate any structural heart abnormalities. The baseline ECG was unremarkable. On the basis of genetic testing, the boy was recommended to remain under regular observation by cardiologists. We ascertained allele frequencies of the *RYR2* variants in a collective of 288 unrelated individuals of Kazakh background (96 Breast cancer patients, 192 healthy control individuals) and only identified variant p.V1810L in an unrelated female breast cancer patient (Table 2). Seven common RYR2 single nucleotide polymorphisms with varying allele frequencies in ethnic groups (Table 3) were tested among the VT study group and yielded comparable frequencies. Intermediate allele frequencies for individual SNPs are a result of the heterogeneous demographic background of the VT study group.

**Table 2:** Frequency of RYR2 single nucleotide variants.

Variant	KazVTSG	ESP6500	1000 Genomes	96 KazBCSC	192 KazCG	HGMD
T2504M	0.0286	Neg.	Neg.	Neg.	Neg.	CM010424
V1810L	0.0286	Neg.	Neg.	0.0104	Neg.	Neg.
D4631V	0.0286	Neg.	Neg.	Neg.	Neg.	Neg.

KazVTSG: Kazakh Ventricular Tachycardia Study Group (n = 35). KazBCSC: Kazakh Breast Cancer Study Cohort (n = 96). KazCG: Kazakh control group (n = 192). HGMD: Human Genome Mutation Database. ESP6500, the 1000Genomes and the 192 KazCG are negative for the observed RYR2 variants. Variant p.V1810L was detected in 1/96 unrelated female breast cancer patient from the KazBCSC.

**Table 3:** Allele frequencies of common RYR2 single nucleotide polymorphisms in various study populations.

SNP-ID		rs10754602		rs16835237		rs3765097		rs147479514		rs2253273		rs2253831		rs790889	
		A	T	C	T	C	T	C	G	G	A	C	T	C	T
KazVTSG		0.700	0.300	0.186	0.814	0.357	0.643	0.971	0.029	0.957	0.043	0.343	0.657	0.400	0.600
HapMap-CEU	European	0.619	0.381	0.102	0.898	0.403	0.597	n.a.	n.a.	0.951	0.049	0.307	0.693	0.327	0.673
HapMap-HCB	Asian	0.561	0.439	0.302	0.698	0.186	0.814	n.a.	n.a.	0.860	0.140	0.222	0.778	0.709	0.291
HapMap-JPT	Asian	0.648	0.352	0.192	0.808	0.203	0.797	n.a.	n.a.	0.860	0.140	0.125	0.875	0.715	0.285
ESP 6500	EA	0.560	0.440	0.099	0.901	0.394	0.606	0.988	0.012	0.958	0.042	0.308	0.692	0.339	0.661
ESP 6500	All	0.527	0.473	0.088	0.912	0.488	0.512	0.991	0.009	0.851	0.149	0.274	0.726	0.327	0.673
1000G	EA	0.594	0.406	0.118	0.882	0.421	0.579	0.989	0.011	0.958	0.042	0.290	0.710	0.332	0.668
1000G	All	0.572	0.429	0.145	0.855	0.457	0.543	0.989	0.011	0.823	0.177	0.219	0.781	0.432	0.568
1000G	Asian	0.670	0.330	0.265	0.735	0.276	0.724	1	0	0.858	0.142	0.180	0.820	0.691	0.309
1000G	European	0.593	0.406	0.118	0.882	0.420	0.580	0.989	0.011	0.958	0.042	0.290	0.710	0.332	0.668

To date, no genotype-based risk stratification or differential treatment approach has been suggested based on CPVT-positive variants. However, early genetic testing is recommended by the Heart Rhythm Society and the European Heart Rhythm Association for clinical management and therapeutic decisions involving family members, because CPVT may present as SCD or Sudden Infant Death Syndrome as the first manifestation [13], [14], [18]. It is a life-saving challenge to ascertain whether and how the effect of low-penetrance variants is modeled by the genetic background or non-genetic factors (life-style, environmental stressors, etc.). Especially a close clinical follow-up of low-penetrance variant carriers will essentially contribute to the understanding of further risk factors and their impact on disease onset and progression.

## Supporting Information

File S1 **Supporting Tables and Figures.** This file contains Table S1, Table S2, and Figure S1- Figure S3. Table S1, Patient data and medical history. Table S2, RYR2 oligonucleotide sequences. Figure S1, Electrocardiogram of Case #271. Figure S2, Electrocardiogram of Case #239. Figure S3, Electrocardiogram of Case #444.
(DOCX)
