# Supplementary material for: RYR2 Sequencing Reveals Novel Missense Mutations in a Kazakh Idiopathic Ventricular Tachycardia Study Cohort
Source: PLoS One. 2014 Jun 30;9(6):e101059. doi: 10.1371/journal.pone.0101059 (PMC4076244; doi:10.1371/journal.pone.0101059)
Supplement: File S1 — Supporting Tables and Figures. This file contains Table S1, Table S2, and Figure S1- Figure S3. Table S1, Patient data and medical history. Table S2, RYR2 oligonucleotide sequences. Figure S1, Electrocardiogram of Case #271. Figure S2, Electrocardiogram of Case #239. Figure S3, Electrocardiogram of Case #444. (DOCX) [file pone.0101059.s001.docx]

**File S1: Supporting Tables and Figures**

Table S1: Patient data and medical history.

| № | ID | Age at disease onset | Diagnosis | Sex | Nationality | Family History of SCD | Treatment/Therapy |
| --- | --- | --- | --- | --- | --- | --- | --- |
| 1 | 243 | 7 | VT | Female | Kazakh | No | - |
| 2 | 291 | 1 | VT | Female | Kazakh | No | - |
| 3 | 239 | 13 | CPVT | Female | Kazakh | Syncope,  No SCD | RFA of RV 2009, ICD 2009 |
| 4 | 266 | 13 | VT | Male | Russian | No | RFA 2012 |
| 5 | 267 | 30 | VT | Female | Kazakh | No | RFA 2011 |
| 6 | 271 | 40 | VT | Male | Korean | No | Diaphragmal hernia plastics 2010 |
| 7 | 295 | 40 | VT | Male | Russian | No | ICD |
| 8 | 308 | 48 | VT | Female | Russian | No | ICD 2000 |
| 9 | 315 | 41 | CPVT | Female | Russian | No | ICD |
| 10 | 317 | 57 | VT | Male | Kazakh | No | ICD, AV node ablation 2012 |
| 11 | 318 | 60 | VT, AF | Male | Korean | No | ICD 2012 |
| 12 | 319 | 49 | VT | Male | Kazakh | No | Coronary bypass 2012 |
| 13 | 226 | 58 | VT, AF | Male | Ukrainian | No | ICD, 2012 |
| 14 | 279 | 25 | VT | Male | Kazakh | No | ICD, 2012 |
| 15 | 280 | 24 | VT | Female | Kazakh | No | ICD, 2012 |
| 16 | 347 | 46 | VT | Female | Russian | No | ICD 2013 |
| 17 | 354 | 71 | VT | Female | Lithuanian | No | ICD 2013 |
| 18 | 357 | 22 | VT, AF | Male | Kazakh | No | RFA 2011, 2013 |
| 19 | 365 | 38 | VT | Male | Kazakh | No | RFA 2013 |
| 20 | 408 | 49 | VT | Male | Russian | No | EPS 2013 |
| 21 | 415 | 25 | VT | Female | Russian | No | No |
| 22 | 427 | 10 | VT | Male | Russian | No | RFA 2013 |
| 23 | 444 | 19 | VT | Female | Kazakh | No | RFA 2013 |
| 24 | 460 | 9 | VT | Male | Kazakh | No | EFS 2013 |
| 25 | 471 | 29 | VT | Female | Kazakh | No | No |
| 26 | 477 | 10 | VT | Male | Russian | No | RFA 2013 |
| 27 | 465 | 6 | VT | Male | Kazakh | No | RFA 2013 |
| 28 | 487 | 27 | VT | Male | Russian | No | RFA 2013 |
| 29 | 488 | 55 | VT | Male | Kazakh | Yes  Father - SCD | ICD 2013 |
| 30 | 491 | 30 | VT | Female | Kazakh |  | ICD 2013 |
| 31 | 456 | 41 | VT | Male | Russian | No | ICD 2013 |
| 32 | 387 | 22 | VT | Female | Kazakh | No | No |
| 33 | 442 | 45 | VT | Female | Kazakh | No | RFA 2013 |
| 34 | 321 | 53 | VT | Female | Russian | No | - |
| 35 | 490 | 55 | VT | Female | Kazakh | No | Renal denervation 2013 |
| Abbreviations: VT – ventricular tachycardia, CPVT – catheholaminergic paroxysmal ventricular tachycardia, AF – atrial fibrillation, SCD – sudden cardiac death, RFA – radiofrequency ablation, RV – right ventricle, ICD – implantable cardioverter defibrillator, AV node – atrioventricular node, EPS – electrophysiological study. | | | | | | | |

Figure S1: Electrocardiogram of Case #271


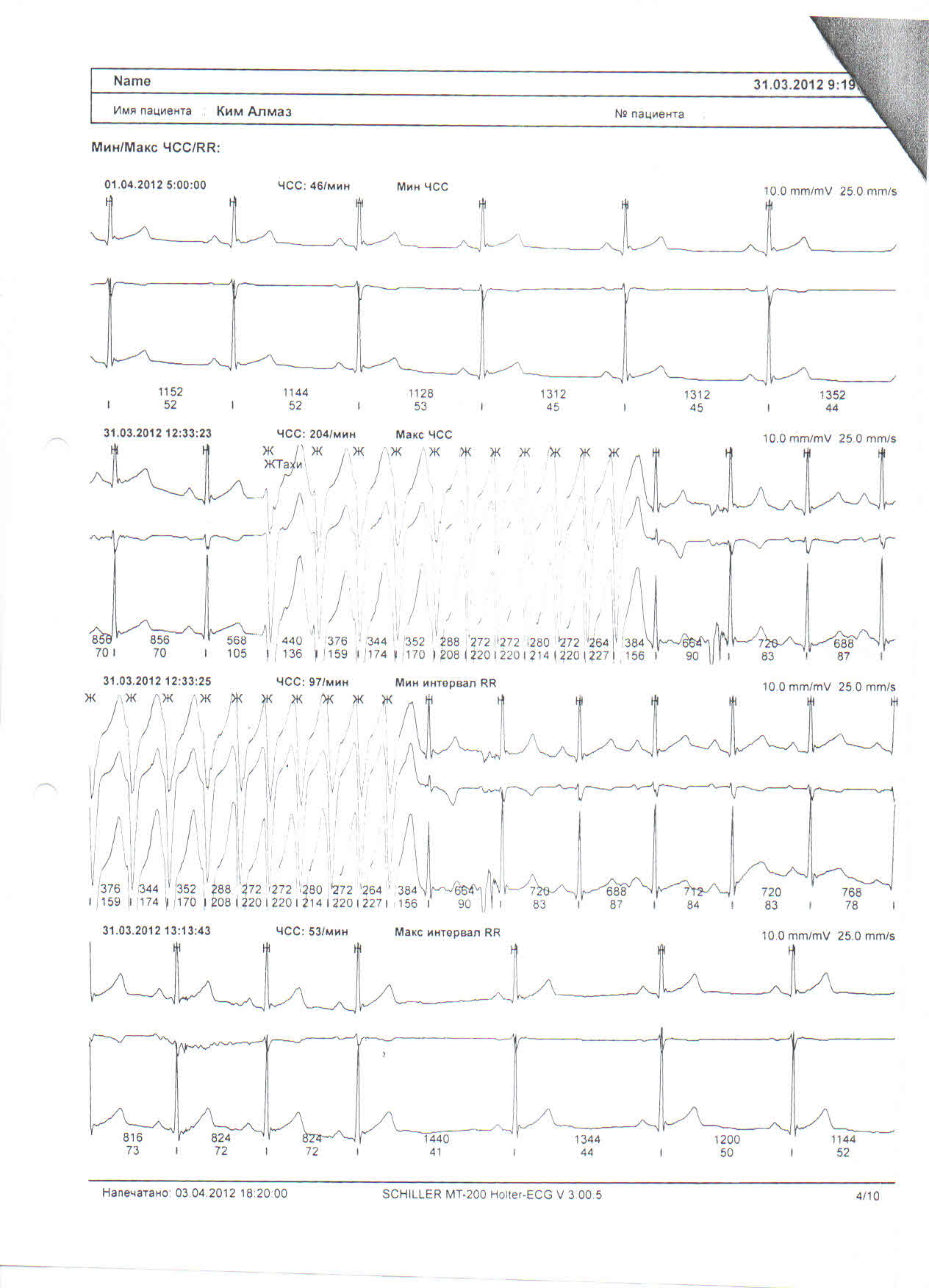


Wide QRS-complex tachycardia with elements for diagnosis of VT (AV dissociation), probably originating in LV. CL is changing depicting warming-up phenomenon (non-reentrant mechanism).

Resting ECG

The ECG heart rhythm is sinusoid with 49 beats/min, a normal QRS axis, a normal PR interval (188ms), QRS (74) ms and QT (446 ms), QTC (402 ms). There were no ST-T or T wave abnormalities.

VT characteristics

At the time of VT onset, the preceding sinus rate at CPVT onset was 83 beats/min, bigeminy. The CPVT heart rate was 220 beats/min, CL - 227 ms. By the duration onset was non sustained and consisted of 14 complexes, duration of paroxysm was 3,2 sec. The VT was monomorphic.

Figure S2: Electrocardiogram of Case #239


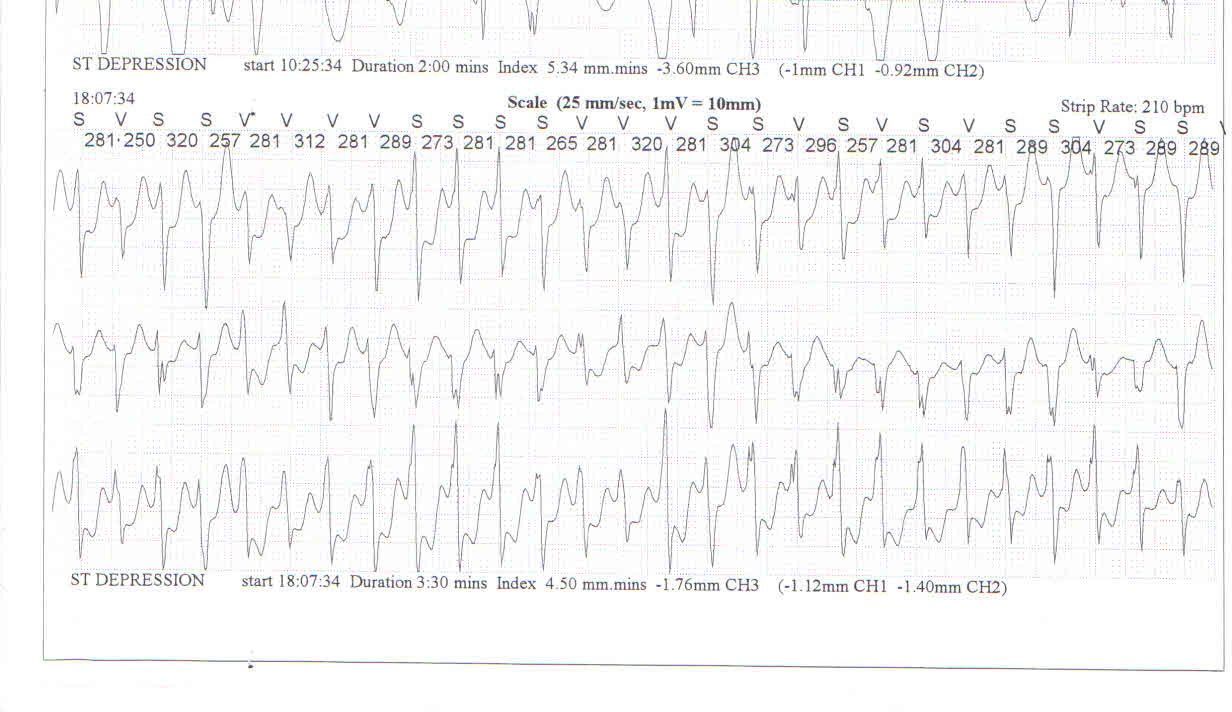


An episode of sustained wide QRS-complex tachycardia, regular RR-intervals (210 bpm) and varying morphology.

Resting ECG

The ECG heart rhythm is sinusoid with 60 beats/min, a normal QRS axis, a normal PR interval (166ms), QRS (96) ms and QT (432 ms), QTC (428 ms). There were no ST-T or T wave abnormalities. The arrhythmias detected at rest were premature ventricular beats, atrial tachycardia.

VT characteristics

At the time of VT onset, QRS complex was relatively narrow, at 90 ms. The preceding sinus rate at CPVT onset was 119 beats/min., bigeminy. The CPVT heart rate was 234 beats/min, CL - 260 ms. Patient had many onsets of CPVT during 2009-2012, electrophysiological characteristic was similar. The CPVT was polymorphic and bidirectional.

The QRS morphology is characteristic of bidirectional ventricular tachycardia changed every 5-6 beats, typically with inferior and superior axis or right bundle branch block and left bundle branch block patterns. CPVT was induced by exercise. Exercise test was not performed. Catecholamine infusion test was not performed.

Figure S3: Electrocardiogram of Case #444


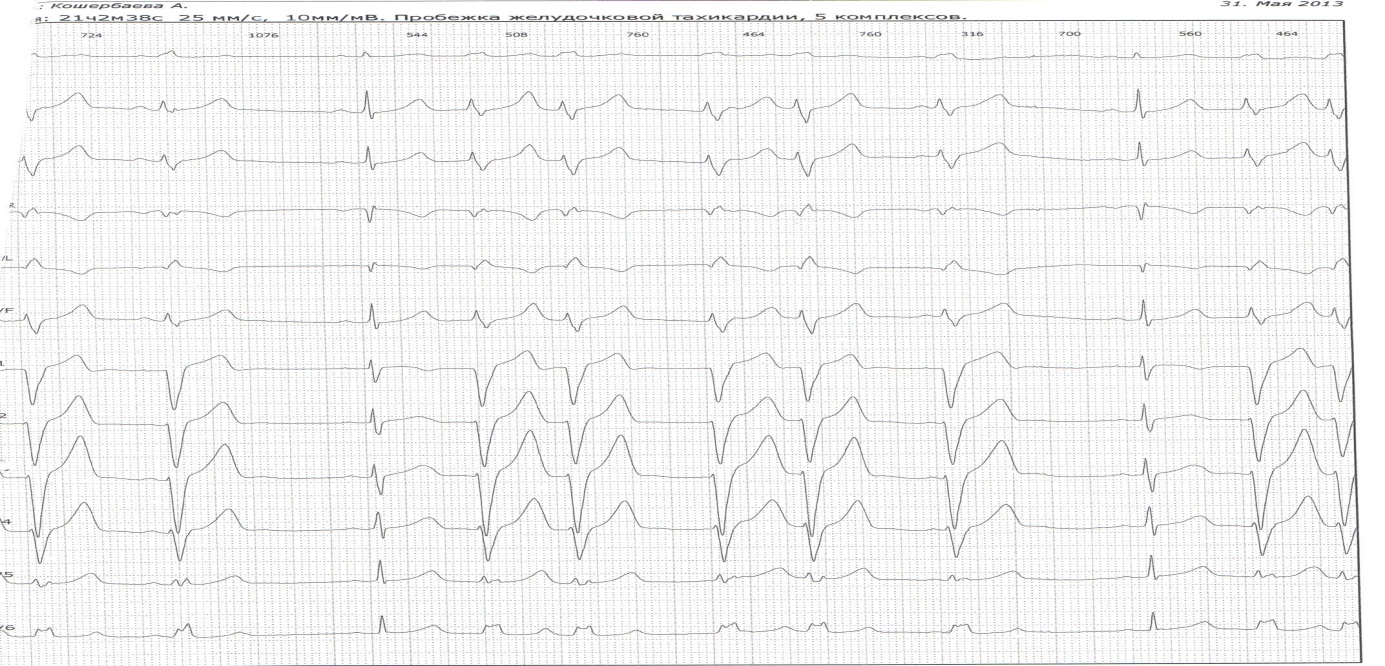


The ECG showed non sustained ventricular tachycardia with morphology of LBBB.

Resting ECG

The ECG heart rhythm at rest was sinusoid with 76 beats/min.  The electrical heart axis was at vertical position. PR (140) ms, QRS (86) ms, QT (382 ms), QTC (398 ms). The ST-T waves were normal.

VT characteristics

24-hour ECG Holter monitoring revealed 6642 ventricular episodes with morphology of LBBB, where PVBs 600, bigeminy PVBs 332, paired PVBs 1544, paroxysms of VT 192 with maximum of 5 complexes.

Table S2: RYR2 oligonucleotide sequences.

| Oligo sequence (5´>3´direction) | OligoID |
| --- | --- |
| **GTAAAACGACGGCCAGTG**TGATGCTGCTGACTGCTCTT* | RYR2E3fw |
| **GAAACAGCTATGACCATG**ATTCCAACTGAAAGGCATGG | RYR2E3rv |
| GTAAAACGACGGCCAGTGGAAAGTTGTGTGTTGGGAATCA | RYR2E8fw |
| GAAACAGCTATGACCATGTTTGCACCCACATCACATTA | RYR2E8rv |
| GTAAAACGACGGCCAGTGTGTCTGGCTATCAGCACCTG | RYR2E10fw |
| GAAACAGCTATGACCATGTGATGCTACTGGCCTGAATG | RYR2E10rv |
| GTAAAACGACGGCCAGTGGTGATTGTGGGTGCTATTGG | RYR2E12fw |
| GAAACAGCTATGACCATGTGGAAGAGAAACTGGGAATTT | RYR2E12rv |
| GTAAAACGACGGCCAGTGGGCCATTATTTCAGGGGACT | RYR2E13fw |
| GAAACAGCTATGACCATGGAATCTGCCCTTTCACTTGC | RYR2E13rv |
| GTAAAACGACGGCCAGTGAGTCCCCTGTACCTGCCTTT | RYR2E14fw |
| GAAACAGCTATGACCATGTGCATATCCAATAAAGAAATGAAAA | RYR2E14rv |
| GTAAAACGACGGCCAGTGCCATGATCACTGCTTTTGGA | RYR2E15fw |
| GAAACAGCTATGACCATGAGGCCTGTTTCTCCTGTTCA | RYR2E15rv |
| GTAAAACGACGGCCAGTGTCCAATATTTTAGGGCTGCAT | RYR2E17fw |
| GAAACAGCTATGACCATGCGAAGCAAAGAAAAATAGGAGTG | RYR2E17rv |
| GTAAAACGACGGCCAGTGGAGGGAGGATCAGCTGAAAG | RYR2E19fw |
| GAAACAGCTATGACCATGTCAGCTACAGGGCAAGAACA | RYR2E19rv |
| GTAAAACGACGGCCAGTGTGACTTTGGCTCTGAAGCTG | RYR2E21fw |
| GAAACAGCTATGACCATGCTTCAAGGCACAGGCAAAAT | RYR2E21rv |
| GTAAAACGACGGCCAGTGTGAGGTAGGGCCAGAAAATG | RYR2E26fw |
| GAAACAGCTATGACCATGTCCAAATGTGCACTATATGTGATCT | RYR2E26rv |
| GTAAAACGACGGCCAGTGAGCCCTTGGTATTGCTTTGA | RYR2E27fw |
| GAAACAGCTATGACCATGCATCATCTTGCAACCGAAGA | RYR2E27rv |
| GTAAAACGACGGCCAGTGCTGGTCTGACAGTGGCTGAT | RYR2E28fw |
| GAAACAGCTATGACCATGGATGACTGGCACAGGACAAA | RYR2E28rv |
| GTAAAACGACGGCCAGTGTGCGTGCTGGCTACTATGAC | RYR2E37fw1 |
| GAAACAGCTATGACCATGCCATTACACAGGAATGCAAAGA | RYR2E37rv1 |
| GTAAAACGACGGCCAGTGTTTTTCCTTCAAATTTACAGTGC | RYR2E37fw2 |
| GAAACAGCTATGACCATGTCCAGTGGGAACTCTGGACT | RYR2E37rv2 |
| GTAAAACGACGGCCAGTGTTTATGAGGGCTGGTAAGCAA | RYR2E40fw |
| GAAACAGCTATGACCATGTCAGCAGTTGGGTCATTCTG | RYR2E40rv |
| GTAAAACGACGGCCAGTGTTGGGGGTACAGGATATGGA | RYR2E41fw |
| GAAACAGCTATGACCATGCATTGTGCACATGTACCATAAAA | RYR2E41rv |
| GTAAAACGACGGCCAGTGGGTTGGACACATGGTTGGAT | RYR2E42fw |
| GAAACAGCTATGACCATGGAGTGAGCCTCTGTCTCCAAA | RYR2E42rv |
| GTAAAACGACGGCCAGTGGTGCCAGGCTTGATTTTCAT | RYR2E43fw |
| GAAACAGCTATGACCATGTCAATTTGCACTTTGCTGATG | RYR2E43rv |
| GTAAAACGACGGCCAGTGTTTTGTATGGAGTTTATAGTTACAGCA | RYR2E44fw |
| GAAACAGCTATGACCATGCAATGACTTCTGCACCAACC | RYR2E44rv |
| GTAAAACGACGGCCAGTGTGAGTTTTCAGCCAAGGGATA | RYR2E45fw |
| GAAACAGCTATGACCATGTTCCTTCATGAAAGTGCTGAGA | RYR2E45rv |
| GTAAAACGACGGCCAGTGTGGCTTTATTAGTATATTGTTGGGTTT | RYR2E46fw |
| GAAACAGCTATGACCATGTTCCAGCACCAAATTCCATT | RYR2E46rv |
| GTAAAACGACGGCCAGTGTTCTCCCTTAAAAACGTCAAGC | RYR2E47fw |
| GAAACAGCTATGACCATGTGGTTTTGGATGCTGTTATGC | RYR2E47rv |
| GTAAAACGACGGCCAGTGATTCGCAGGTAAGCCAGAAA | RYR2E48fw |
| GAAACAGCTATGACCATGGTGGAGAGGTTGGGATGTTG | RYR2E48rv |
| GTAAAACGACGGCCAGTGGCCATTGACACCAAAATTCA | RYR2E49fw |
| GAAACAGCTATGACCATGACCCATGGCTTACCTGAAAA | RYR2E49rv |
| GTAAAACGACGGCCAGTGCCCCATGTTAATCCCTTTGA | RYR2E50fw |
| GAAACAGCTATGACCATGGAACTTGACTGGGAGCAAGG | RYR2E50rv |
| GTAAAACGACGGCCAGTGAAACAAGTTGCCTCGTGAAAA | RYR2E75fw |
| GAAACAGCTATGACCATGGGGACAAATATAAATTCAAAAGTTGC | RYR2E75rv |
| GTAAAACGACGGCCAGTGTGCTTCGAGGTGTGTTCCTA | RYR2E83fw |
| GAAACAGCTATGACCATGGAAAACAAGACACTGCCGATT | RYR2E83rv |
| GTAAAACGACGGCCAGTGGAAAGCATTTGCCTTTGGAG | RYR2E86fw |
| GAAACAGCTATGACCATGAACAGTGGCTGTTTTGCTCT | RYR2E86rv |
| GTAAAACGACGGCCAGTGGGCATAAAGTCCAAGACTGGTT | RYR2E87fw |
| GAAACAGCTATGACCATGTGCCAAATGGTGAAGCAAT | RYR2E87rv |
| GTAAAACGACGGCCAGTGTCTCCTTCTTTCACTTTGAGTTCC | RYR2E88fw |
| GAAACAGCTATGACCATGTTTGCTGATGATTACAGTCTCCA | RYR2E88rv |
| GTAAAACGACGGCCAGTGTTTATTGCCAGAGCAGCATC | RYR2E89fw |
| GAAACAGCTATGACCATGCTGATTCCATAAGCCCATGC | RYR2E89rv |
| GTAAAACGACGGCCAGTGGGGACATATCCTTGATTCAGATG | RYR2E90fw1 |
| GAAACAGCTATGACCATGTTGTACCTGAGCGCAAACAG | RYR2E90rv1 |
| GTAAAACGACGGCCAGTGTGGTGGATATGCTTGTGGAA | RYR2E90fw2 |
| GAAACAGCTATGACCATGCGTTCTGGCACTAGCATGAA | RYR2E90rv2 |
| GTAAAACGACGGCCAGTGGGCTCCCTCAATTCATTCAA | RYR2E91fw |
| GAAACAGCTATGACCATGTTCTGTGGCTCCTTGACAAT | RYR2E91rv |
| GTAAAACGACGGCCAGTGGGCAGAAATGTTCTCCCTCA | RYRE93fw |
| GAAACAGCTATGACCATGGCCTAGGCACCAGTATTTCA | RYRE93rv |
| GTAAAACGACGGCCAGTGAGAGGGCTTCCCCACAGTAT | RYRE94fw |
| GAAACAGCTATGACCATGCCAGCTCAGAATCTGCTTCC | RYRE94rv |
| GTAAAACGACGGCCAGTGAGTGACCACAAGATATGCCAGT | RYR2E95fw |
| GAAACAGCTATGACCATGTGCAGGAACTCCAACCAAAT | RYR2E95rv |
| GTAAAACGACGGCCAGTGTGATGTTAGCCAAATTCATTGT | RYR2E96fw |
| GAAACAGCTATGACCATGCACATCAAATAAAAATCTAAACTGTGC | RYR2E96rv |
| GTAAAACGACGGCCAGTGTGGTTGAAGCCAACAAAATG | RYR2E97fw |
| GAAACAGCTATGACCATGTTCTGACATTGAAGGAAAGGAAA | RYR2E97rv |
| GTAAAACGACGGCCAGTGTCTGTTCCTGGCTTATTTTGC | RYR2E99fw |
| GAAACAGCTATGACCATGGCAGACCCTCCCTTCAAAAA | RYR2E99rv |
| GTAAAACGACGGCCAGTGCGGCTGTGTTCTCACTAGAGC | RYR2E100fw |
| GAAACAGCTATGACCATGAAACAGCCCTGCAACTTTTT | RYR2E100rv |
| GTAAAACGACGGCCAGTGTGGGTAATGTCCCTCCAAGA | RYR2E101fw |
| GAAACAGCTATGACCATGGCTTTGCCAGGAAACACTTG | RYR2E101rv |
| GTAAAACGACGGCCAGTGCCATGTTCTGAGCATTTTGC | RYR2E102fw |
| GAAACAGCTATGACCATGCTCCTCCTTCCCACATTCAT | RYR2E102rv |
| GTAAAACGACGGCCAGTGCAGCCTCCATGTGATGATCT | RYR2E103fw |
| GAAACAGCTATGACCATGTGGAGAGGTGGAAGATTCTGA | RYR2E103rv |
| GTAAAACGACGGCCAGTGAAATTTGCATGTGGCGTTTT | RYR2E104fw |
| GAAACAGCTATGACCATGTGCCTCACTGTTTAATGCACA | RYR2E104rv |
| GTAAAACGACGGCCAGTGTGTTTTGTTAGCACACACTTTGG | RYR2E105fw |
| GAAACAGCTATGACCATGTGCTCCCAGAAAACACACAA | RYR2E105rv |
| *- Forward and reverse primers contain a M13 forward or reverse sequence as highlighted in bold for the first primer pair. | |
